# Sugar-Lowering Drugs for Type 2 Diabetes Mellitus and Metabolic Syndrome—Strategies for In Vivo Administration: Part-II

**DOI:** 10.3390/jcm8091332

**Published:** 2019-08-28

**Authors:** Raquel Vieira, Selma B. Souto, Elena Sánchez-López, Ana López Machado, Patricia Severino, Sajan Jose, Antonello Santini, Amelia M. Silva, Ana Fortuna, Maria Luisa García, Eliana B. Souto

**Affiliations:** 1 Department of Pharmaceutical Technology, Faculty of Pharmacy, University of Coimbra (FFUC), Pólo das Ciências da Saúde, 3000-548 Coimbra, Portugal; 2 Department of Endocrinology, Braga Hospital, Sete Fontes, 4710-243 São Victor Braga, Portugal; 3 Department of Pharmacy, Pharmaceutical Technology and Physical Chemistry, Faculty of Pharmacy and Food Sciences, University of Barcelona, Institute of Nanoscience and Nanotechnology (IN2UB), Av. Joan XXIII, 27-31, 08028 Barcelona, Spain; 4 Centro de Investigación biomédica en red de enfermedades neurodegenerativas (CIBERNED), 28031 Madrid, Spain; 5 Laboratory of Nanotechnology and Nanomedicine (LNMED), Institute of Technology and Research (ITP), Av. Murilo Dantas, 300, Aracaju 49010-390, Brazil; 6 Department of Pharmacy, University of Tiradentes (UNIT), Industrial Biotechnology Program, Av. Murilo Dantas 300, Aracaju 49032-490, Brazil; 7 Department of Pharmaceutical Sciences, Mahatma Gandhi University, Cheruvandoor Campus, Ettumanoor, Kerala 686631, India; 8 Department of Pharmacy, University of Naples Federico II, Via Domenico Montesano, 49-80131 Naples, Italy; 9 Department of Biology and Environment, University of Trás-os Montes e Alto Douro (UTAD), Quinta de Prados, 5001-801 Vila Real, Portugal; 10 Centre for Research and Technology of Agro-Environmental and Biological Sciences (CITAB-UTAD), Quinta de Prados, 5001-801 Vila Real, Portugal; 11 Laboratory of Pharmacology, Faculty of Pharmacy, University of Coimbra (FFUC), Pólo das Ciências da Saúde, 3000-548 Coimbra, Portugal; 12 CIBIT—Coimbra Institute for Biomedical Imaging and Translational Research, University of Coimbra, 3000-548 Coimbra, Portugal; 13 CEB—Centre of Biological Engineering, University of Minho, Campus de Gualtar, 4710-057 Braga, Portugal

**Keywords:** diabetes mellitus, animal models, in vivo, administration routes

## Abstract

Diabetes is a complex disease characterized by hyperglycemia, together with polyuria, polydipsia, and polyphagia. While Type 1 diabetes mellitus (T1DM) results from genetic, environmental, or immune dysfunction factors leading to pancreatic β-cell destruction depriving the organism from endogenous insulin, Type 2 diabetes mellitus (T2DM) is characterized by peripheral insulin resistance. Depending on the type of diabetes mellitus and drug mechanism to study, the animal model should be carefully selected among the wide variety of the currently available ones. This review discusses the most common animal models currently employed to study T1DM and T2DM. Moreover, an overview on the administration routes that could be used is also discussed.

## 1. Introduction

Diabetes mellitus (DM) is one of the current leading health problems worldwide. DM comprises a group of metabolic diseases characterized by hyperglycemia, as insulin production by pancreatic β-cells is either insufficient or even absent, and target cells do not respond to circulating insulin [1,2]. Associated with hyperglycemia, together with common symptoms, namely polyuria, polydipsia, and polyphagia [1], DM is a silently life-threatening condition that may culminate on hemodynamic and cardiovascular complications, metabolic complications, and altered genetic susceptibility [3]. Also, long-term developing diseases, as a consequence of hyperglycemia affecting the whole organism or being organ specific, as is the case of diabetic retinopathy, is in the natural course of the disease [4].

DM is classified into two main types: type 1 DM (T1DM), previously known an insulin dependent DM (IDDM), and type 2 DM (T2DM), previously known as non-insulin dependent DM (NIDDM) [5]. In addition, gestational DM is characterized by the development of DM by pregnant women with hyperglycemia during pregnancy with no previous history of the disease [3]. T1DM results from a complex disease process in which genetic, environmental factors, and/or immune dysfunction lead to an autoimmune response, resulting in pancreatic β-cell destruction, depriving the organism from endogenous insulin [6,7]. T2DM comprises about 85% of DM cases and is characterized by peripheral insulin resistance. Compensatory insulin hypersecretion from pancreatic β -cells precedes the decline in islet secretory function. Reduced insulin sensitivity affects mainly the skeletal muscle, liver, and adipose tissue, as these tissues have particular requirements for glucose uptake and metabolism [3,7].

In general, DM consequences affect various systems, organs, and tissues [4,8,9,10,11], being therefore difficult to predict in the exact consequences of the disease, as genetic, nutrition, environment, and other factors are different in each individual [10,11]. With these constraints, several animal models have been developed, aiming at assessing the causes and consequences of DM, in order to achieve effective and safe treatments. This review describes the animal models currently available to study DM and its complications. Additionally, since the administration routes are of crucial relevance in order to study their effectivity, a comprehensive overview about the different administration routes used for pre-clinical and clinical trials has been undertaken. In this sense, the key to successful drug development relies on the suitable selection of the animal model and the drug administration route.

## 2. Diabetes Mellitus Animal Models

The study of DM comprises the study of normal individuals, as only by knowing how a physiological organ/tissue responds to stimuli can we correlate to the altered response in compromised organ/tissues. The study of glucose regulated insulin secretion, as well as its modulation by oral antidiabetic-drugs, has been made with resource to albino mice (Charles Rivers breeding), using isolated islets of Langerhans [12,13] or isolated pancreatic β-cells [14,15]. Other sources of pancreatic β-cells from physiological donors have also been used, such as porcine, human, canine, among others [13,14,15].

Animal models may be developed by two principal mechanisms: disease induction (e.g., using specific drugs) or genetic manipulation. Both are of extreme relevance as they allow the study of specific disease-related mechanisms and are essential to understand the pathogenesis and progression of the diseases and extrapolate to humans.

Since T1DM and T2DM are metabolic disorders, representing complex integrating bodily systems, choosing the adequate animal model to use in specific in vivo experiments requires careful consideration [16]. To attain this objective, a careful analysis must be performed when choosing a DM animal model, regarding particular aspects of the disease and the specific knowledge that is aimed in each study [16]. In this context, T1DM animal models include spontaneously developing autoimmune diabetic animals, or animals that have been subjected to chemical ablation of pancreatic β-cells. On the other hand, T2DM animal models are more numerous, and may embrace obese and non-obese models, with variable insulin resistance and β-cell failure degrees [16]. In addition, transgenic and knock-out mouse models are also available, but their use in the research field is still controversial, as it will be herein focused [16].

The models most commonly used to study T1DM and T2DM are summarized in Table 1 and Table 2, respectively, together with their main advantages and disadvantages. In an idealistic perspective and aiming a reliable representation of the diversity observed among diabetic human patients, at least two or more animal models should be employed, taking into account the principles of the four Rs, i.e., replacement (preference for methods that avoid or replace animals use), reduction (use methods which minimize the number of animals per experiment), refinement (prefer methods regarding a minimal animal suffering and that promote their welfare), and responsibility [16].

## 3. Diabetes Mellitus Type 1 Animal Models

T1DM is mainly characterized by pancreatic β-cells autoimmune destruction, which contributes to insufficient insulin production, or even to the absence of insulin secretion [16]. In animal models of T1DM, this deficiency may be reproduced by several mechanisms, ranging from β-cells destruction either by chemical ablation using normal animal models to breeding animals (mostly rodents) that suffer from spontaneously developed autoimmune diabetes [16,38,39]. Some of the most commonly used models of T1DM are outlined in Table 1 and are mainly constituted by rodent models, but some higher animals, such as pigs, dogs, and primates, have also been used (Table 1).

### 3.1. Chemical Induction of Diabetes Mellitus Type 1

#### 3.1.1. Streptozotocin (STZ)-Induced Models

**Figure 1:**
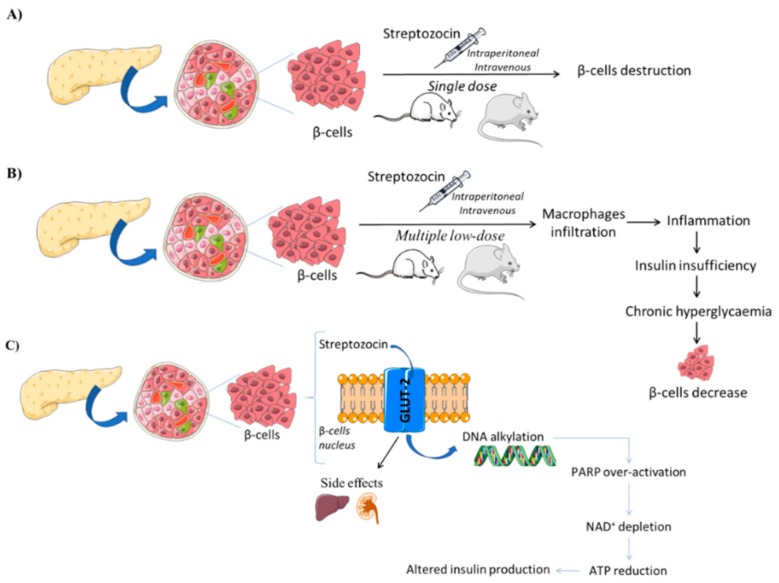
Streptozocin diabetes induction model (based on [17]). (**A**) Single-dose Streptozocin, (**B**) Multiple-low dose streptozocin, (**C**) Streptozocin mechanism on the β-cells nucleus and side effects in other organs with glucose transporter subtype 2 (GLUT-2) receptors. Streptozotocin (STZ) behaves as a glucose analogue and is transported into the pancreatic β-cell by GLUT-2. It produced DNA alkylation and over-activation of poly-ADP ribose polymerase (PARP) causing NAD+ depletion, cellular ATP reduction, and compromising insulin.

Streptozotocin (STZ), chemically known as *N*-(methylnitrosocarbamoyl)-α-d-glucosamine, is a naturally occurring compound produced by Streptomycetes achromogenes with antibiotic properties that are selectively taken up by pancreatic β-cells, causing its destruction [16,40]. After its intraperitoneal or intravenous administration, STZ behaves as a glucose analogue and is transported mainly by the glucose transporter subtype 2 (GLUT-2) [41] into the pancreatic β-cell, where it induces toxicity, mainly by producing DNA alkylation [16]. Due to DNA strand breaks, over-activation of poly-ADP ribose polymerase (PARP) leads to NAD+ depletion, cellular ATP reduction, and consequently, insulin production is compromised, as well as the cell survival, since there is a massive loss of energy [42]. As STZ enters the cell via GLUT-2, this is also expressed in other cells behind those from the pancreas, as the toxic action of STZ is not specific to pancreatic β cells, occurring also in hepatocytes and kidney cells (Figure 1) [43]. These are probably the reasons underlying the high mortality rate associated with this model.

Depending on the severity of the intended model disease, STZ-induced DM models usually result from one of the following procedures: administration of a single high STZ dose, or as multiple low STZ doses [16]. In the high-dose STZ administration procedure, a single STZ dose is administered to mice (100–200 mg·kg^−1^) or rats (35–65 mg·kg^−1^) by intravenous or intra-peritoneal routes, producing massive pancreatic β-cell destruction with little or no insulin production [43]. Insulinemia must be recorded to ensure that the intended model is stable, since there is some evidence that pancreatic islets regeneration may occur after this single-dose treatment. On the other hand, multiple low-dose STZ administration implies that small doses (20 to 40 mg·kg^−1^·day^−1^) are to be administered over a period of time in order to promote insulitis [16,18]. During insulitis development, infiltration of macrophages in the pancreatic islet promote cytokine production-dependent T1DM development. Therefore, therapies which target cytokines and nitric oxide are highly probable to be successful in reducing diabetes development in this model. 

Both STZ-induced diabetes models are cheaper and easier to perform than the remaining models and they can be used in most strains of rodents, or other animals, opening the field of diabetes research to an array of genotypic and phenotypic options that would otherwise be inaccessible [18,43].

#### 3.1.2. Alloxan-Induced Models

Alloxan (2,4,5,6(1H,3H)-pyrimidinetetrone, 2,4,5,6-tetraoxypyrimidine,5,6-dioxyuracil) is also used to chemically induce DM and two possible mechanisms have been proposed. One suggests that alloxan selectively inhibits glucose-induced insulin secretion through specific inhibition of glucokinase, the pancreatic beta cell glucose sensor [44], and it also induces reactive oxygen species (ROS) formation, creating a redox cycle generating superoxide radicals. Alloxan is reduced to dialuric acid and then re-oxidized back to alloxan, producing superoxide radicals that undergo dismutation (by superoxide dismutase) to form hydrogen peroxide; concomitantly, hydroxyl radicals may also be formed by side reactions. These highly reactive oxygen species may cause β-cell DNA fragmentation, leading to apoptosis [16,44,45]. Although alloxan is also taken up by the liver, hepatotoxicity induced by alloxan is minimal or null since the liver has more effective protection mechanisms against ROS than β-cells [45], and they also have several mechanisms for xenobiotic biotransformations and elimination. Alloxan also promotes the essential –SH groups oxidation, especially that of gluthatione (GSH), enzymes, and proteins, and also dysregulates intracellular calcium homeostasis, leading to supraphysiological calcium concentrations and, hence, cellular damage [16,44,45]. 

**Figure 2:**
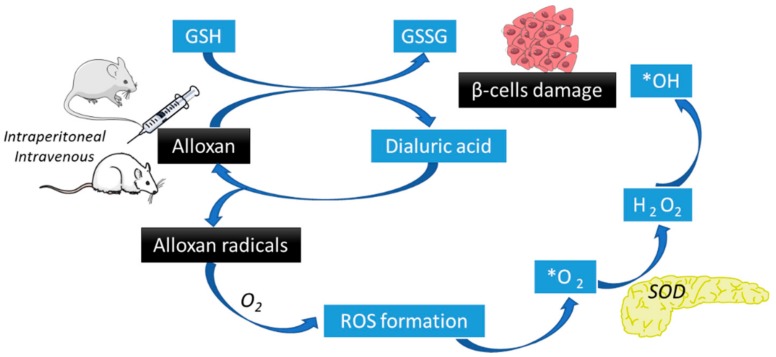
Alloxan induced diabetes mechanism (based on [46]). Alloxan is reduced to dialuric acid and re-oxidized to alloxan producing alloxan radicals and reactive oxygen species (ROS) which undergo dismutation (by superoxide dismutase, SOD) to form hydrogen peroxide (H_2_O_2_). Hydroxyl radicals (*OH) may also be formed by side reactions. These *OH cause β-cell DNA fragmentation, leading to apoptosis.

Alloxan administered doses range from 50 to 200 mg/kg (in mice) and 40 to 200 mg/kg (in rats), and they are dependent on the chosen strain and the route of administration (e.g., intraperitoneal and subcutaneous alloxan administration require doses up to three times of those administered by intravenous route) [16]. Alloxan induces DM models as a consequence of ROS mediated beta cell toxicity (Figure 2), allowing the investigation and understanding of ROS mediated mechanisms of beta cell death in both T1DM and T2DM [44].

### 3.2. Spontaneous or Autoimmune of Diabetes Mellitus Type 1

#### 3.2.1. Non-Obese Diabetic Mice

The non-obese diabetic (NOD) mouse model originated in the inbreeding of the Cataract Shionogi (CTS) strain in the 1980s. The NOD mouse model is an autoimmune disease model where the T1DM develops spontaneously [21]. NOD mice exhibit polyuria, glycosuria, weight loss, and lymphocytic infiltration of the islets of Langerhans within the pancreas. It has been observed in NOD mice that innate immune cells infiltrate the pancreas of NOD mice at 3 weeks of age. In the same way, these cells types, such as dendritic cells, macrophages, and neutrophils, are also found in the human islet infiltrate [47]. The infiltration of innate immune cells into the islets attract adaptive CD4 and CD8 T cell subsets, which are required for DM development, into the islets from approximately 4 to 6 weeks of age [21,46].

Additionally, the relevance of this model relays in the fact that it was able to identify key genetic and environmental risk factors, such as effects of microorganisms including the gut microbiota, and how they may contribute to T1DM [21]. 

#### 3.2.2. Biobreeding Rats

The biobreeding (BB) rat model was developed in the 1970s from outbred Wistar rats [21]. The incidence of diabetes is the same in male and female BB rats, although in humans it is reported to have a slight prevalence in males [23]. The BB rats develop T cell-specific lymphopenia and an impairment of Treg cell function. At 5 weeks, the BB rats develop insulitis, followed by hyperglycemia in around 70% of the animals [23]. Once hyperglycemia occurs, BB rats manifest polyuria, leading to a severe loss of body weight despite excessive drinking. Afterwards, these rats will develop ketoacidosis within several days [23]. According to Medina and colleagues, in this model there is a deterioration of beta cell function and mass as well as intra-islet blood flow that precedes insulitis and diabetes [24]. These underlying changes in islet function may be previously unrecognized factors of importance in type 1 diabetes development [24].

#### 3.2.3. LEW.1AR1/-Iddm Rats

The LEW.1AR1-iddm rat is an animal model T1DM, which arose through a spontaneous mutation in the Dock8 gene within the major histocompatibility complex (MHC) congenic background strain LEW.1AR1 [48]. This Dock8 mutation provides a deepened insight into the impact of genes involved in diabetes development [48]. The mutation leads to a variable T-cell frequency in peripheral blood. The secondary lymphatic organs, such as lymph nodes and spleen, show 15% less T cell frequency, whereas the frequency of β-cells increased by 10% in the lymph nodes and by 5% in the spleen [49].

According to Arnd and colleagues, the Dock8 mutation is responsible for changed immune cell frequencies in different compartments, together with the RT1B/Du haplotype autoimmune diabetes [49].

### 3.3. Genetically Induced Diabetes Mellitus Type 1

#### AKITA Mice

One of the most widely used genetically induced diabetic mouse models is AKITA mice. These mice possess a mutation in Ins2, causing abnormal folding of insulin [48,49]. This model involves chronic stress on protein processing, involving the endoplasmic reticulum and unfolded protein response, triggering apoptosis and diabetes. The unfolded protein response tries to compensate and reduces the protein load of the endoplasmic reticulum, increasing its folding capacity [50]. This leads to toxicity in pancreatic β cells, decreasing their insulin secretion. Hyperglycemia is found in males, but it is attenuated in female mice [49]. In comparison with STZ induction, AKITA mice develop more robust alterations in albuminuria and kidney structure [49].

### 3.4. Virally Induced Diabetes Mellitus Type 1

Viruses have been observed as a factor that could be implicated in the pathogenesis of DM1 [51]. In this sense, different viruses are able to induce T1DM, such as Coxsakie virus, encephalomyocaridits virus, Kilham rat virus, lymphocytic choriomeningitis virus rubella, and mumps (see Table 1). These viruses stimulate autoreactive T cells that participate in islet destruction by triggering β-cell-specific autoimmunity that end up in destruction of the β-cells [51].

### 3.5. Non-Rodent Models of Diabetes Mellitus Type 1

#### 3.5.1. Pancreatomy

Pancreatomy is a reliable but invasive method to induce hyperglycemia and has been used in pigs and primates [16]. However, this method leads to pancreatic exocrine deficiency in the animal [16]. Moreover, partial pancreatomy can be combined with STZ treatment [16].

#### 3.5.2. Chemical Ablation of β-Cells in Large Animals

Chemical ablation of beta cells in large animals could be undertaken using STP or alloxan, as previously explained. However, it has the main drawback of interspecies variation requiring different dosages, which can cause variated effects [16].

## 4. Diabetes Mellitus Type 2 Animal Models

Briefly, T2DM is mainly characterized by hyperglycemia due to insulin resistance and pancreatic β-cell insufficient compensating, leading to metabolic impairments for several organs and tissues, namely, adipose and muscular tissues. To study the onset, progress, and effective treatments for T2DM complications, several animal models have been established in order to investigate the mechanisms and pathophysiology of T2DM. Thus, these animal models may include models of insulin resistance or β-cell failure, but also models that develop diseases related to hyperglycemia exposure. Animal models of T2DM can be divided into artificially induced diabetic models, spontaneously diabetic models, and transgenic/knock-out diabetic models. Independently of the T2DM animal model type, the majority of the animals are obese, with several degrees of obesity, modeling, hence the close association of obesity and T2DM development, similar to the metabolic syndrome [16,52]. 

In this respect, obese models can be monogenic or polygenic. Most monogenic models have a defect in the signaling of leptin, which is the key regulator of energy balance, body adiposity, and glucose homeostasis. Leptin signaling was shown to be compromised in metabolic diseases, such as obesity and diabetes mellitus, corroborating the relevant role of this hormone in the etiology and pathophysiological manifestations of those conditions [52]. Some monogenic animal models with compromised leptin signaling are described in Table 2. 

Polygenic models of obesity provide a more reliable model of the human condition, and, therefore, efforts have been made to construct models in which several genes have been altered or even deleted. Thus, a great variety of these models have been employed to study obesity, glucose intolerance, and DM, since they also allow a great variety of genotypes to be assessed [16,53,54]. These models have been used to study the correlation between obesity and insulin resistance, to study the possible reversion of T2DM symptoms, to correlate obesity and glucose homeostasis, aiming, in general, a better understanding of T2DM and associated complications. 

Some of the most commonly used models of T2DM are described in Table 1 and Table 2, as some models can be used to treat both types of DM. In particular, some additional considerations about KK mice (obese polygenic model) and high-fat feeding models will be mentioned next.

### 4.1. Obese Monogenic Models of Diabetes Mellitus Type 2

Genetic manipulation can also identify genes of interest in the development of diabetes. Monogenic models are useful in understanding the connection between how insulin resistance develops in an obese phenotype [55]. In this sense, the most common models are discussed below.

#### 4.1.1. Lep^ob/ob^ Mice

The Lep^ob/ob^ mice were deficient in leptin, leading to an increased body weight and hyperinsulinaemia [55]. Hyperglycemia appears at 4 weeks of age and increases until around 4 months. Islet mass is increased, and insulin secretion is maintained in this model in addition to reduced metabolic rate, dysregulation of thermogenesis, hyperlipidemia, and infertility [55].

#### 4.1.2. Lepr^db/db^ Mice

These mice have an autosomal recessive mutation in the Ob-Rb leptin receptor [55]. These animals present hyperphagia, obesity, hyperinsulinemia, and hyperglycemia. Interestingly, the background mice possess a strong effect on the severity of the phenotype, e.g., on a C57Bl/KSJ, where the animals develop severe ketogenesis and have a short life span, whereas on C57BL/6 mice, these symptoms are considerably milder [55].

#### 4.1.3. Zucker Diabetic Fatty (ZDF) Rats

The Zucker fatty rats were discovered from the simple autosomal recessive (fa) gene on chromosome after a cross of Merck M-strain and Sherman rats in 1961 as a model for human obesity and T2D [51]. These rats possess a hypothalamic defect in leptin receptor signaling, which is associated with type IV hyperlipidemia and hypertension. These rats develop proteinuria and glomerulosclerosis, ultimately leading to renal failure [51]. In some cases, abnormal glucose tolerance is also reported, due to the metabolic defects in the hepatic organ [51].

### 4.2. Obese Polygenic Models of Diabetes Mellitus Type 2

There are a variety of different polygenic models of obesity. These may more accurately reflect human disease, which is generally polygenic. However, one disadvantage of these models is that they have no wild-type control [56].

#### 4.2.1. KK Mice Models

KK mice strain derives from wild-derived ddY mice, which are obese and hyperleptinemic. This model develops severe hyperinsulinemia and insulin resistance (in both muscle and adipose tissue). Total pancreatic insulin content is increased, leading to hypertrophic and degranulated pancreatic islets. This strain also develops diabetic nephropathy signs, increased glucose, and hemoglobin HbA1c levels, and impaired glucose tolerance [16,57]. As several breeding colonies of these mice have been maintained in several laboratories, there are now several substrains, such as T-KK (or Toronto-KK, KK/Upj), KK/HlLt, KK/Ta, and KK/San [57]. A substrain of the KK model, the KK-AY mice, was created by inserting a yellow obese AY gene (mice develop yellow coat instead of dark), an introgression of the AY mutation in an agouti gene. This strain develops maturity-obesity, more severe hyperinsulinemia, and more prominent pancreatic islets alterations, mainly due to the ectopic expression of the agouti protein which functions as a melanocortin receptor 4 (MCR4) antagonist in the hypothalamus, which has increased HbA1c and exhibits early stage nephropathy [16,57,58].

#### 4.2.2. Otsuka Long-Evans Tokushima Fat (OLEFT) Rat

The Otsuka Long-Evans Tokushima Fat (OLEFT) rat was derived from a spontaneous obesity in an outbred colony of Long Evans rats in the 1990s [59]. These rats show a deficit in the cholecystokinin (CCK)-1 receptor gene, resulting in the absence of CCK-1 receptors in the gastrointestinal track and the brain. OLETF rats have increased expression of hypothalamic neuropeptide Y (NPY), thus contributing to the hyperphagia developed by these animals. The study of OLETF rats has roles of the NPY in energy balance and glucose homeostasis [59].

#### 4.2.3. New Zealand Obese (NZO) Mice

New Zealand obese (NZO) mice is a model of obese mice due to hyperphagia and reduced expense of energy [32]. NZO mice develop insulin resistance, hypertension, and hypercholesterolemia. Hyperglycemia and hyperinsulinemia are also found at early ages, and they are associated with β-cell destruction [32]. Gender differences have been observed in this model, with males presenting increased rates of T2DM [32].

#### 4.2.4. TallyHo/Jng Mice

TallyHo mice were developed by Jackson laboratory from the progeny of diabetic males [60]. Obesity and reduced insulin sensitivity may be key features for diabetes in Tally-Ho mice [60]. They develop hyperglycemia between 10 and 14 weeks of age. Also, they show hyperinsulinemia, hyperlipidemia, moderate obesity, and enlargement of the islets of Langerhans [60]. 

There are marketed gender differences, since females do not show DM, despite displaying moderate hyperinsulinemia, hyperlipidemia, and obesity [60].

#### 4.2.5. NoncNZO10/LtJ Mice

NoncNZO10/LtJ mice were generated by combining New Zealand obese (NZO/HlLt) mice and non-obese non-diabetic (NON/ShiLtJ) mice at the Jackson laboratory [61]. NoncNZO10/LtJ mice are not hyperphagic and do not show hypercorticism, and no thermoregulatory defects [62]. At 8 weeks, male mice develop insulin resistance and increased hepatic glucose production that leads to obesity and T2DM at around 13 weeks of age [62]. These animals show early islet hypertrophy, followed by β-cell degradation and β-cell atrophy [62].

### 4.3. Induced Obesity Models of Diabetes Mellitus Type 2

#### 4.3.1. High Fat Feeding Models

It is known that high fat feeding contributes to the development of obesity, hyperinsulinemia, and glucose homeostasis impairment, since there is insufficient compensatory action by pancreatic islets, resulting in impaired glucose tolerance and weight gain [63]. To create this model, the normal diet (consisting of about 26% protein, 63% carbohydrate, and 11% fat) given to C57BL/6J mice was replaced by a diet in which the number of calories from fat is substantially increased, contributing to around 58% of total calories daily consumed [63]. During the establishment of this model, a strict monitoring of the daily eaten food is essential, in order to ensure that mice are not eating less than usual [16]. This model developed several characteristics of human T2DM symptoms—hyperglycemia, hyper-insulinemia—followed by a decline in insulin secretion and weight gain [63]. Ultimately, some attention should be paid to the strain’s background when attempting to induce a model of T2DM, as it may determine the susceptibility of intended metabolic alterations induced by diet. 

#### 4.3.2. Dessert Gerbil

Diabetes in Dessert gerbil rats (*Psammomys obesus*) was discovered by chance observation in desert rodents collected by the US naval medical research unit in Egypt in the 1960s [64]. They are characterized by muscle insulin resistance and the incapacity of insulin to activate insulin signaling on a high energy diet. This leads to hyperglycemia and hyperinsulinemia, resulting in beta cell failure and increased proinsulin secretion [64]. On a high energy diet, the animals did not persist for more than a few months [65].

#### 4.3.3. Nile Grass Rats

The Nile grass rat (NGR), Arvicanthis niloticus, is an herbivorous African murine rodent [66]. Fed a conventional lab diet, NGRs spontaneously develop obesity, hyperglycemia, and hypertension [66]. However, these rats do not develop diabetes in the wild, but it is induced under laboratory conditions [67]. The disease is manifested in various organs, such as lipid deposition in the liver, advanced glycation end product (AGE) deposits in the kidney, and beta-cell failure [67].

Since there are a wide range of animal models available in order to study DM, a careful choice of the animal models pretended for experimental use should be performed, attending to the exact purpose of the study. Thus, aiming at developing successfully experiments using the least possible number of animals, the choice of an animal model for DM study should respond to the initial investigational question. If T1DM is the target of the study, the required autoimmunity and the timing and predictability of onset (variable among strains) must be considered, as well as the islet cell destruction mechanisms. For T2DM, the mechanisms underlying hyperglycemia, in non-obese and in obese models, such as the associated pathologies (e.g., atherosclerosis, dyslipidemia) and other complications, namely diabetic nephropathy and neuropathy, must be taken into account. Furthermore, subjects’ gender, genetic phenotype, and environmental background are also relevant to the study. 

In addition to the animal model, in order to obtain robust and reproducible results, a suitable drug administration should be performed. The route has to take into account the drug’s physicochemical parameters, sample volume, animal safety, and also be able to extrapolate results into the clinical practice. Administration of substances to the different diabetic animal models requires careful consideration and planning to optimize delivery of the agent to the animal, while minimizing potential adverse experiences from the procedure.

## 5. Administration Routes

When preparing a pharmacokinetic experiment using laboratory animals, together with the preparation of the animal before drug administration (feeding versus fasting), the appropriate administration route, site of administration, and sample volume should be adequately set. The administration routes that require less manipulation techniques are recommended [68]. In animal experimentation, several routes for substance administration are currently available, namely enteral (through the digestive tract), either oral (into the mouth) or gavage (esophageal, gastric, nasogastric or orogastric), intravenous (into a blood vessel), epicutaneous (onto the skin), intradermal (into the skin), subcutaneous (under the skin), transdermal (across the skin), intramuscular (into a muscle), transcorneal (onto the eye), intraocular (into the eye), intracerebral (into the brain), epidural (into the dura mater surrounding space), intrathecal (into distal spinal cord surrounding space), intraperitoneal (into the peritoneal cavity), intraosseous (into the marrow cavity), intranasal (sprayed into the nose and then absorbed by the nasal mucous membranes or into the lungs), intratracheal (into the lungs by direct tracheal instillation), inhalation, and other less common techniques, such as those using other body natural orifices, surgical exposure, and others regarding species-specific anatomic features [68,69,70,71,72]. 

### 5.1. Enteral Administration

The enteral administration route comprises techniques in which the substance is delivered through the digestive tract, such as addition of drugs to drinking water, to the food, intragastric administration through oral gavage or rectal administration [68,73,74]. This route is very economical, practical, and considered as safe, depending on the compound being tested. However, it presents some limitations: (i) slower onset of action, (ii) potentially significant first-pass effect by intestine and liver, (iii) lack of systemic absorption from the digestive tract, (iv) poor efficacy, (v) poor compliance when voluntary administered, and (vi) the impossibility of using the procedure in unconscious subjects or in individuals with clinically relevant diarrhea or emesis. Special concerns must be taken when administering a large volume administration by orogastric or nasogastric gavage, since it may lead to stress by promoting gastric distension in species that cannot vomit (e.g., rodents); smaller volumes (~5 mL/kg) are preferable [68]. This administration route is mainly preferable for single administrations or short-period administrations, as longer administrations may lead to animal stress or discomfort. When animals avoid food or beverage because of a drug’s flavor or smell, administration may be done using oral gavage [73,74]. This route opens a window in order to aid the development of enteral nutrition formulas, such as the recent study published by Mesejo and colleagues which developed a high-protein diabetes-specific formula that reduces insulin needs and improves glycemic control [75]. Moreover, nutraceuticals can also be explored as a new strategy in order to treat diabetes.

### 5.2. Intravenous Administration

This route is quite efficient and advantageous, since drugs do not suffer the first-pass-effect by the liver, and bioavailability is 100% [68]. This route is convenient when: (i) a rapid drug effect is required, (ii) continuous administration (infusion) is intended, (iii) large volume dosage drug is needed; and (iv) other routes provides are not appropriate [68,73]. The choice of administration site depends greatly on the animal, such as the jugular vein in the neck (e.g., large animals, rats) and tail vein (e.g., mice, rats) [53]. Special attention should be paid to drugs pharmacokinetics, individual maximum tolerated dose, dosing intensity, and minimal variation in peak and blood concentrations required. The procedure requires aseptic preparation of the skin for percutaneous venous injection, ensuring that the substance is sterile and aseptically delivered [68,76]. A long-term intravenous administration should be closely monitored to avoid, specially, pulmonary edema [68,73,76]. On the clinical practice, this route is applied for emergencies, such as Diabetic ketoacidosis, hyperglycemic hyperosmolar state, and hypoglycemia, which are serious complications of diabetes mellitus [77].

### 5.3. Intraosseous Administration

Intraosseous administration is mainly used for crystalloid fluids delivery, as an alternative to the intravenous route in hypovolemic individuals (whose veins are inaccessible or collapsed) and also in human pediatric medicine [78]. The medullary cavity veins allow the substance to enter directly into the blood flow, although it is difficult to perform and is potentially invasive, and animals are usually anesthetized [68,78]. This route is rarely used for diabetes preclinical or clinical studies.

### 5.4. Dermal Administration

The administration of drugs using the skin rout may be divided into: (i) topical application, i.e., drugs are intended to treat local skin infections (e.g., genital mucosas [79]), burns, inflammation, and wounds, and drugs are absorbed across the epidermis through paracellular and transcellular mechanisms, but drug concentration in the blood is negligible; (ii) transdermal application, in which drugs are applied and absorbed through the skin or mucosal membranes instead of by oral or injectable routes, they are intended to treat areas of the body away from the site of application (e.g., hormone replacement therapy using patches); (iii) intradermal application (drug is deposit into the dermis using a needle, usually forming a bubble) and; (iv) subcutaneous administration (drug is injected in between the skin and muscle) are convenient routes to insulin administration (e.g., [80]). With drug delivery through the skin, in animal models, one must keep in mind the following important factors: surface area, substance concentration, lipid solubility, skin contiguity, skin thickness (at the application site), contact duration, skin hydration, surface occlusion, and overlying hair [48]. Reports obtained using this route focus on the improvement of wound healing of diabetic patients [81,82].

### 5.5. Muscle Administration

Intra-muscular administration comprises a parenteral route commonly used for large animals and humans, since they have a great muscle mass. The rich muscle vascular supply allows a homogeneous and rapid substance absorption, when compared to subcutaneous route, where smaller substance volumes are administered and a more complex technique is required [68]. Some considerations to keep in mind are: avoidance of irritating compounds or accidental nerve injection, which may lead to paresis, paralysis, localized muscle fiber destruction, and even necrosis [83,84]. New therapies are recently being explored using this route, such as the intramuscular administration of human placenta-derived mesenchymal stromal-like cells for patients who have a diabetic foot ulcer with peripheral arterial disease [85].

### 5.6. Epidural and Intrathecal Administration

When a prompt effect on cerebrospinal tissues or meninges is desired, testing substances may be administered either into the epidural or subarachnoid (also named intrathecal) spaces of the spinal cord, thus avoiding the usual absorption restrictions of the blood–brain barrier (BBB) [68]. It is commonly used to induce spinal anesthesia and to visualize vertebral bodies, for example, in contrast imaging studies. It requires an aseptic preparation of the skin and a sterile technique, beyond a perfect knowledge of species variability, epidural fat, substance lipophilicity, injectable leaks through intervertebral spaces, and meningocerebral ligament’s individual anatomy, which may interfere with the product administration and even cause some adverse effects [68,86].

### 5.7. Intraperitoneal Administration

Intraperitoneally applied drug forms are supposed to be located in the peritoneal cavity. Intraperitoneal injection is mostly performed in small species (e.g., rodents) to overcome the challenging intravenous, subcutaneous, and intramuscular access or difficulties, as well as to administer large volumes of substances, to give treatment to the peritoneum cavity, and to perform some surgical procedures [68,83]. Drug pharmacokinetics is similar to that observed after the oral route, due to prior absorption into the mesenteric vessels, subsequent drainage into the portal vein, and thus being subjected to hepatic metabolism before reaching systemic circulation. When performed in mammals, depending on the species, several procedures are adopted to avoid visceral damage by the needles [83] and by injected drugs [87]. The injected products should be sterile, isotonic, and nonirritating in order to avoid complications such as ileus, peritonitis, and adhesions. This is one of the most widely administration routes, especially in rodent models [88,89]. Limitations are the sensitivity of the tissue to irritating substances and lesser tolerance to solutions of non-physiological pH. This route is not applied for diabetes treatment (although it is applied for treatment of other pathologies) to larger mammals or humans, in which intravenous access is easily performed.

### 5.8. Intranasal, Intratracheal, and Inhalational Administration

In the less commonly used administration routes, intranasal, intratracheal, and inhalational routes, drugs are delivered through the respiratory system to access surrounding or system targets. Animals are usually sedated or anesthetized, in order to reduce struggling and sneezing, and small relative volumes are delivered to reduce the risk of suffocation and death [68]. Intranasal delivery may be applied either to local or systemic delivery of nonirritating substances, as nasal mucosa is rich in blood vessels, there is a rapid absorption of the product and a rapid systemic effect, since it has avoided the first-pass effect by the liver [68]. Intranasal administration is becoming a common method to deliver therapeutic drugs to the central nervous system (CNS), as it is non-invasive and allows large molecules that do not cross the BBB to access the CNS, with reduced systemic exposure and unwanted systemic side effects [90]. In turn, intrapulmonary delivery may be performed by (1) intratracheal instillation, an easier but not so effective technique which involves small volumes injected directly into the trachea, or (2) inhalation, a highly complex technique that typically uses vapors or aerosols of nebulized particles in solutions which are deposited by gravitational sedimentation, inertial impaction, or diffusion in small airways; the ones deposited in large airways are then incorporated into the mucus and expelled by the mucocilliary clearance. It is thus important to evaluate solvent and propellant effects, since evaporation may lead to particle size changes [68,89]. Among these, intranasal has shown increased relevance, due to the direct brain connection and to the development of new pharmaceutical formulations able to enhance penetration through the nasal mucosa [90,91].

## 6. Conclusions

Diabetes mellitus is a metabolic disorder characterized by several disturbances in carbohydrate, protein, and fat metabolism and, apart from the need to discover new and more effective anti-diabetic drugs, animal models cannot be avoided in pre-clinical research. Several animal models are available according to the type of diabetes and also with the aim of therapy to assess. In this sense, animal models should be carefully chosen in order to fully reproduce the mechanisms and pharmacokinetics of the proposed therapies. Moreover, drug administration is of extreme relevance, both in the preclinical and clinical stages. Especially during preclinical studies, administration route advantages and drawbacks should be taken into account and the route should be adequate in order to reproduce human pathology and suitable treatment for further clinical trials.

## Figures and Tables

**Table 1:** Animal models most commonly used to study type 1 diabetes mellitus.

Induction Mechanism	Model	Main Features	Possible Uses	Advantages	Disadvantages	Ref.
**Chemical induction**	**High single-dose streptozotocin (STZ)** *	Simple model of hyperglycemia	Testing drugs (new insulin formulations) or therapies (transplantation)	A more stable model	Mortality is relatively more frequent	[17,18]
				Comparatively cheaper, easier to develop and maintain		
	**Multiple low dose streptozotocin (STZ)** *	Model of induced insulitis	Treatments that may prevent β-cell death	Used for longer experimental studies	May produce toxic effects on other tissues	
				May be applied to higher animals	STZ is relatively unstable and solution should ideally be made immediately prior to injection	
	**Alloxan** *	Simple model of hyperglycemia	Transplantation models	Selective loss of pancreatic β-cells leaving α and δ cells intact	Hyperglycemia develops primarily by direct cytotoxic action on the β-cells and insulin deficiency rather than consequence of insulin resistance	[19,20]
				Animals live longer without insulin treatment (since there is a residual insulin secretion)	Less stable and reversible because of the spontaneous regeneration of β-cells	
				Relatively less ketosis and resulting mortality	May produce toxic effects on other tissues	
				Comparatively cheaper, easier to develop and to maintain	High variability of results on development of hyperglycemia	
**Spontaneous autoimmune**	**Non-obese diabetic (NOD) mice** *(Spontaneous autoimmune model of choice)*	β-cell destruction due to an autoimmune process	Understanding genetics of T1DM	Hyperlipidemia can be also studied, as lipid content increase	Polyphagia and polyuria occurrence	[16,21,22]
	**Biobreeding (BB) rats**		Understanding mechanism of T1DM	Hyperglycemia persists for several days	A diabetes and obesity symptom overlaps	[23,24]
	**LEW.1AR1/-iidm rats**		Treatments that may prevent β-cell death and/or manipulate autoimmune process			
					Not identical to those in human disease	
**Genetically induced**	**AKITA mice** *	β-cell destruction due to ER stress. Insulin dependent.	New formulations of insulin Transplantation models Treatments to prevent ER stress	The lack of β-cell mass makes it an alternative to STZ-treated mice in transplantation studies		[25,26]
**Virally-induced**	**Coxsackie B virus**	β-cell destruction induced by viral infection of b-cells	Establish potential role of viruses in the development of T1DM	Stable and irreversible diabetes can be induced	Comparatively costlier to develop	[27]
	**Encephalomyocarditis virus**					
	**Kilham rat virus**					
	**LCMV under insulin promoter**					
					Technical expert is required to handle of virus	
**Non-rodent models**	**Pancreatectomy**	Hyperglycemia induction in pigs, dogs and primates	Treatments that may prevent β-cell death Transplantation models	Reasonably accurate model of auto transplantation of islets in humans	Very invasive surgery	[28]
					In large animal models, spontaneous diabetes is relatively rare and unpredictable in onset	
	**Chemical ablation of β-cells in large animals**			Some models combine a partial pancre-atectomy with STZ treatment, thus reducing the dose of STZ	Interspecies variation in the β-cell toxicity of alloxan or STZ	[28]
					Narrow window of efficacy.	

* Also used in T2DM research.

**Table 2:** Animal models most commonly used to study type 2 diabetes mellitus.

Induction Mechanism	Model	Main Features	Possible Uses	Advantages	Disadvantages	Ref.
**Obese monogenic models**	**Lep^ob/ob^ mice** (mutated leptin gene)	Obesity-induced hyperglycemia, with hyperphagic, obese, hyperinsulinaemic and hyperglycemic animals	Treatments to improve insulin resistance	Pancreatic islet volume dramatically increased	Infertile mice	[29]
					Metabolic aberrations (hyperlipidemia disturbance in temperature regulation, lower physical activity)	
					Diabetes not particular severe and thus not completely representative of human T2DM	
	**Lepr^db/db^ mice** (mutated leptin receptor gene)		Treatments to improve β-cell function		Ketosis after a few months of age, having a relative short lifespan	[30]
	**Zucker Diabetic Fatty (ZDF) Rats** (mutated leptin receptor gene)			Diabetic complications also develop	Hypertensive rats	[31]
					Females do not develop overt diabetes	
**Obese polygenic models**	**KK mice**	Obesity-induced hyperglycemia	Treatments to improve insulin resistance			
	**Otsuka Long-Evans Tokushima Fat (OLEFT) rat**		Treatments to improve β-cell function	Three stages of histological changes can be observed		[32,33]
	**New Zealand Obese (NZO) mice**		Some models show diabetic complications	Renal complications		
	**TallyHo/Jng mice**			Adiposity, plasma triglycerides, cholesterol and free fatty acid levels are increased	Only male mice develop hyperglycemia	
	**NoncNZO10/LtJ mice**			Indicated for diabetic wound healing studies		
				Nephropathy presence		
**Induced obesity models**	**High fat feeding (mice or rats)**	Obesity-induced hyperglycemia	Treatments to improve insulin resistance	Baboons and humans are genetically, anatomically and physiologically very similar	Handling of baboon is somewhat difficult	[31,33]
	**Desert gerbil**		Treatments to improve β-cell function	Cardiac complications can be studied	Veterinarian is required	
	**Nile grass rat**		Treatments to prevent diet-induced obesity		Costly model containing	
**Non-obese models**	**Goto-Kakizaki (GK) rat**	Hyperglycemia induced by insufficient β-cell function or mass	Treatments to improve β-cell function	Allow the study of β-cell function and diabetic complications	Interstrains variability of the islets morphology and metabolism	[31,33]
			Treatments to improve β-cell survival			
**Genetically induced models of β-cell dysfunction**	**Human islet amyloid polypeptide-expressing (hIAPP) mice**	Amyloid deposition in islets	Treatments to prevent amyloid deposition	Express human IAPP under the insulin promoter, which can form amyloid within the islets for further study	Transgenic mice	[31,33]
			Treatments to improve β-cell function			
		β-cell destruction due to ER stress	Treatments to prevent ER stress		β-cell adaption to increased insulin demand is restricted	
			Treatments to improve β-cell survival			
**Non-rodent models**	**Cat models**	Amyloid deposition in islets	Treatments to improve β-cell function	Islet amyloidosis study	More expensive models	[31,33,34,35,36,37]
		β-cell destruction	Treatments to prevent diet-induced obesity			
	**Old-world non-human primates**			Similarities to human condition		
